# Cis and Trans Effects of Human Genomic Variants on Gene Expression

**DOI:** 10.1371/journal.pgen.1004461

**Published:** 2014-07-10

**Authors:** Julien Bryois, Alfonso Buil, David M. Evans, John P. Kemp, Stephen B. Montgomery, Donald F. Conrad, Karen M. Ho, Susan Ring, Matthew Hurles, Panos Deloukas, George Davey Smith, Emmanouil T. Dermitzakis

**Affiliations:** 1 Department of Genetic Medicine and Development, University of Geneva Medical School, Geneva, Switzerland; 2 Institute of Genetics and Genomics in Geneva (iGE3), Geneva, Switzerland; 3 Swiss Institute of Bioinformatics (SIB), Geneva, Switzerland; 4 MRC Integrative Epidemiology Unit, University of Bristol, Bristol, United Kingdom; 5 School of Social and Community Medicine, University of Bristol, Bristol, United Kingdom; 6 University of Queensland Diamantina Institute, Translational Research Institute, Brisbane, Queensland, Australia; 7 Department of Pathology and Genetics, Stanford University, Stanford, California, United States of America; 8 Wellcome Trust Sanger Institute, Hinxton, United Kingdom; 9 William Harvey Research Institute, Barts and The London School of Medicine and Dentistry, Queen Mary University of London, London, United Kindom; 10 Princess Al-Jawhara Al-Brahim Centre of Excellence in Research of Hereditary Disorders (PACER-HD), King Abdulaziz University, Jeddah, Saudi Arabia; 11 Center of Excellence for Genomic Medicine Research, King Abdulaziz University, Jeddah, Saudi Arabia; Perelman School of Medicine, University of Pennsylvania, United States of America

## Abstract

Gene expression is a heritable cellular phenotype that defines the function of a cell and can lead to diseases in case of misregulation. In order to detect genetic variations affecting gene expression, we performed association analysis of single nucleotide polymorphisms (SNPs) and copy number variants (CNVs) with gene expression measured in 869 lymphoblastoid cell lines of the Avon Longitudinal Study of Parents and Children (ALSPAC) cohort in cis and in trans. We discovered that 3,534 genes (false discovery rate (FDR) = 5%) are affected by an expression quantitative trait locus (eQTL) in cis and 48 genes are affected in trans. We observed that CNVs are more likely to be eQTLs than SNPs. In addition, we found that variants associated to complex traits and diseases are enriched for trans-eQTLs and that trans-eQTLs are enriched for cis-eQTLs. As a variant affecting both a gene in cis and in trans suggests that the cis gene is functionally linked to the trans gene expression, we looked specifically for trans effects of cis-eQTLs. We discovered that 26 cis-eQTLs are associated to 92 genes in trans with the cis-eQTLs of the transcriptions factors BATF3 and HMX2 affecting the most genes. We then explored if the variation of the level of expression of the cis genes were causally affecting the level of expression of the trans genes and discovered several causal relationships between variation in the level of expression of the cis gene and variation of the level of expression of the trans gene. This analysis shows that a large sample size allows the discovery of secondary effects of human variations on gene expression that can be used to construct short directed gene regulatory networks.

## Author Summary

Humans differ in their genetic sequences at millions of positions but only a subset of these differences have a functional effect. In order to detect functional genetic differences, we assessed the impact of common genetic variants on gene expression in 869 individuals and discovered that the expression of many genes is affected by common variants in cis or in trans. We show that the effect of some variants on gene expression cannot be detected in other tissues, highlighting the tissue specificity of gene regulation. In addition, we show that variants associated to common diseases are more likely to affect gene expression in cis and in trans. Finally, we show that variants affecting gene expression in cis often affect gene expression in trans, which suggests that the trans effects are due to the cis genes expression. We tested this hypothesis and discovered several cases of genes regulated in trans by a cis regulated gene in a causal manner. This shows that a population-based strategy with a large number of individuals has the potential to detect secondary effects of common variants that can be used to construct short directed regulatory networks.

## Introduction

Genome-wide association studies (GWAS) have discovered a large number of loci implicated in many complex traits and diseases [1]. The vast majority of variants discovered are found in non-coding regions (88%), which challenges the interpretation of their functional effect [1]. One way to overcome this challenge is to look for associations between variants and an intermediate cellular phenotype, such as gene expression. Expression quantitative trait loci (eQTL) analysis have been successful in mapping variants to gene expression in several cell types providing a better understanding of the genetics of gene expression, and revealing functional impacts of variants associated with complex traits and diseases [2]–[10].

Most studies so far were conducted on relatively small sample sizes [11], limiting the power to detect variants affecting gene expression in cis and to a greater extent in trans, as trans-eQTLs typically have weaker effect sizes than cis-eQTLs [12]. Detecting eQTLs with small effect sizes is indeed important, as a variant can have a weak effect in the tissue sampled but a strong effect in the tissue relevant for a specific disease. Furthermore, small effects in cis can be important if the associated gene plays a substantial role in a cellular process. A large sample size also allows the discovery of variants that are both cis and trans-eQTLs, suggestive of a regulatory relationship between the cis regulated gene and the trans regulated gene.

Here we measured gene expression in Lymphoblastoid cell lines (LCLs) from 869 genotyped individuals of the Avon Longitudinal Study of Parents and Children (ALSPAC) cohort in order to map single nucleotide polymorphisms (SNPs) and copy number variants (CNV) with minor allele frequency >1% to gene expression in cis and in trans. We then investigated trans effects of cis-eQTLs and used causal models to investigate the mechanism by which a variant can affect the expression of a gene in cis and in trans.

## Results

In order to better understand how genetic variations affect gene expression in LCLs, we associated 2'290'057 imputed single-nucleotide polymorphisms (SNPs) from the HapMap2 reference set and 3329 copy number variants (CNVs) to gene expression in 869 individuals from the ALSPAC cohort. We first looked for cis-eQTLs, defined as variants associated with gene expression in a 2 MB window surrounding the transcriptional start site (TSS). We used spearman rank correlation to test for association between 20'745 probes on autosomes, measuring the expression of 14'835 genes, and variants present in the windows. A gene-based significance threshold was determined by permuting all expression phenotypes 1000 times (**Methods**). We discovered that 3534 genes had a cis-eQTL at a false discovery rate (FDR) of 5% (3498 due to SNPs, 36 due to CNVs) (**Table S1, Table S2**). As shown previously [5], [9], we found that cis-eQTLs cluster around the TSS (**Figure S1**). CNVs were more often cis-eQTLs than expected by chance (i.e: if SNPs and CNVs had the same probability to be a cis-eQTL, we would expect the same fraction of CNVs and SNPs in our cis-eQTL results than in the whole data set) (odd ratio: 7.1, Fisher's exact test pvalue<2.2e-16), suggesting that CNVs are more likely to affect gene expression than SNPs. We observed a strong correlation (rho = 0.98, pvalue = 1e-13) between sample size and the number of genes with a cis-eQTL discovered (**Figure S2**). Although, it appears that the rate of discovery is decreasing for large sample sizes, it is likely that further increases in sample size would allow the discovery of more genes with at least one cis-eQTL.

**Figure 1:**
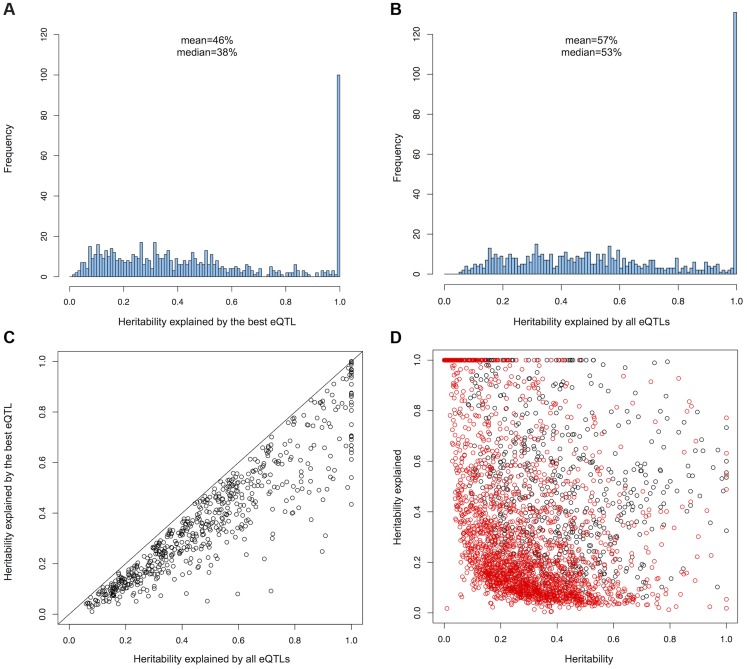
(A) Histogram of heritability explained by the best cis-eQTL and (B) all independent cis-eQTLs for genes with more than one cis-eQTL. (C) Heritability explained by independent cis-eQTLs compared to the heritability explained by their best cis-eQTL for genes with more than one cis-eQTL. (D) Heritability of genes with one cis-eQTL (red) and more than one cis-eQTL (black) compared to heritabilty explained by all available eQTLs.

In order to detect genes affected by more than one cis-eQTL, we performed conditional regression by removing the effect of the cis-eQTL(s) on gene expression and repeating the association analysis on the residuals until no significant associations could be detected (**Methods**) [13], [14]. We found that 694 genes (19.6% of the genes with a cis-eQTL) have at least two independent cis-eQTLs (**Figure S3**). For 374 genes (53.9%), independent cis-eQTLs were located in the same recombination interval, showing that variants in linkage disequilibrium can have different functional effects on gene expression. In order to evaluate the importance of independent cis-eQTLs on the heritability of gene expression, we obtained heritability estimates from the MUTHER cohort in LCLs [2]. On average, the heritability of genes with multiple cis-eQTLs was greater than for genes with only one cis-eQTL detected (mean heritability = 0.24 for genes with one cis-eQTL, mean heritability = 0.38 for genes with multiple eQTLs, Mann-Whitney U test pvalue<2.2e-16). For each gene with multiple eQTLs, we compared the heritability explained by the best eQTL to the heritability explained by all independent eQTLs using a linear model where the standard normal expression of the gene is explained by the best eQTL or all independent eQTLs (**Methods**). We observed that the best eQTL explains on average 46% of the heritability of the traits while all independent eQTLs explain on average 57% of the heritability of gene expression (**Figure 1**). These results show that independent cis-eQTLs are detected preferentially in genes with a relatively high genetic component of their expression and that independent cis-eQTLs explain 11% more of the heritability of the gene expression on average than using only the best cis-eQTL.

**Figure 2:**
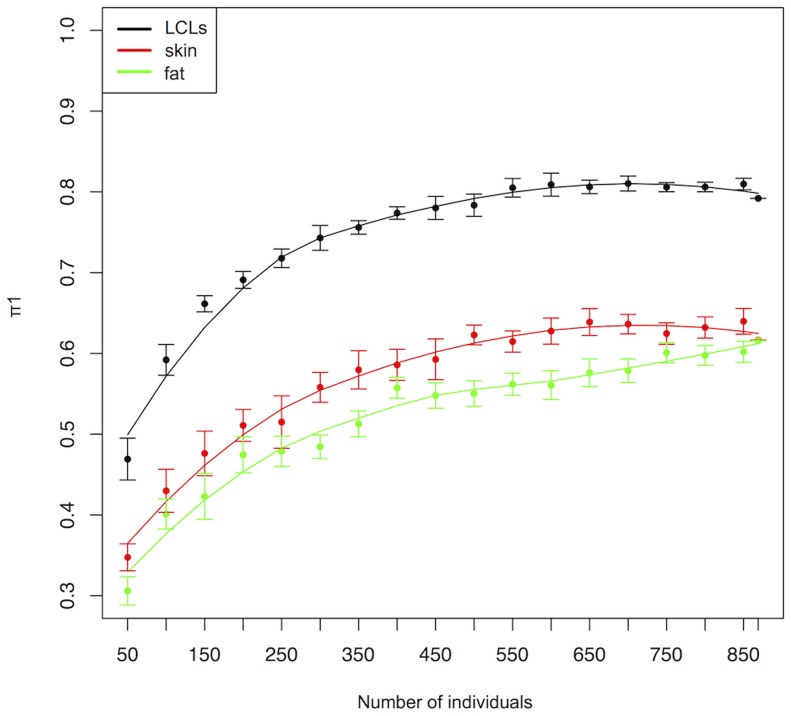
Tissue sharing (π1) of cis-eQTLs between ALSPAC LCLs and cis-eQTLs detected by the MUTHER cohort in LCLs (black), skin (red) and adipose tissues (green) tissues in function of the ALSPAC sample size. Error bars represent the 5% and 95% confidence interval and lines show the best polynomial fit of the data.

Several studies have shown that the effect of variants on gene expression is tissue dependent [5], [9], [10], [15]. Indeed, some eQTLs can have different effect sizes in cells of different developmental origin [9] or can be detected only in specific tissues [5], [9], [10], [15]. However, the estimated tissue sharing of eQTLs has steadily increased in function of the cohort sample sizes, ranging from 20%–31% [5] with a small cohort to 56–83% in a larger cohort [2], questioning the relevance of interrogating different tissues. In order to address this question, we took advantage of the large sample size of the ALSPAC cohort to investigate the effect of sample size on tissue sharing. We obtained cis-eQTLs detected in LCLs, skin and adipose tissues from the MUTHER cohort [2], one of the largest studies investigating eQTL tissue specificity. We assessed tissue sharing as a function of sample size in a continuous manner by matching cis-eQTLs detected by the MUTHER study (1%FDR) with the pvalues detected for the same associations in different subsets of individuals of the ALSPAC cohort and computed the π1 statistic (estimate of the proportion of true positives in a pvalue distribution) for each sample size [16]. We observed little tissue sharing for small sample sizes (30.6% with adipose tissue, 34.8% with skin, and replicated 46.9% in LCLs for 50 individuals) (**Figure 2**). In contrast, using the entire ALSPAC cohort, we replicated 79.2% of the eQTLs in LCLs, detected by the MUTHER project, and estimated tissue sharing to be 61.6% for adipose tissue and 61.7% for skin cells. We did not observe an increase in the π1s for LCL and skin cells after 600 individuals, while the sharing of MUTHER adipose eQTLs with ALSPAC LCLs continued to increase slightly for larger sample sizes (**Figure 2**). We found stronger concordance in the directionality of eQTLs replicating within LCLs (1.8% with opposite directionality at 5% FDR) compared to eQTLs shared across tissues (10.4% with opposite directionality in skin and 10.5% in adipose tissue at 5%FDR). These results indicate that a relatively large proportion of cis-eQTLs detected in one tissue cannot be detected in other tissues and support the idea that one should perform eQTL analysis in different tissues in order to map all regulatory variants in the genome.

We next investigated whether we could detect variants affecting gene expression in trans. We defined trans-eQTLs as variants affecting gene expression at a distance greater than 5 MB from the TSS or on another chromosome. We used spearman rank correlation to test for association between 21'634 probes, measuring the expression of 14'441 genes on autosomes and chromosome X, and all variants further than 5 MB from their TSS. A genome-wide significance threshold was determined by permuting a subset of the expression phenotypes 1000 times (**Methods**). We discarded trans associations of CNVs when the gene associated was on the same chromosome or on a chromosome with SNPs correlated with the CNV (r^2^>0.1) as some CNVs appeared to be mismapped (**Text S1**). In addition, we discarded all significant associations of CNVs with an imbalance in copy number between males and females, as this resulted in the false trans associations of the CNVs with genes differentially expressed between males and females. After filtering, we discovered trans-eQTLs for 48 genes (FDR = 5%) (45 due to SNPs and 3 to CNVs) **(** **Table 1** **, Table S2, Table S3)**. We assessed the replication of the trans-eQTLs using the MUTHER cohort [2], a twin cohort, which we separated in two sets of unrelated individuals (group 1: 340 individuals, group 2: 338 individuals). We replicated 22 trans-eQTLs (of 40 tested) with a pvalue<0.05 in the first group and 19 in the second group (union = 23). In order to validate the array-based trans-eQTLs with an independent technology, we used the GEUVADIS [17] cohort (373 individuals with RNA-seq in LCLs) and replicated 11 trans-eQTLs (of 32 tested). As for cis-eQTLs, CNVs were more often trans-eQTLs than expected by chance (i.e: if SNPs and CNVs had the same probability to be a trans-eQTL, we would expect the same fraction of CNVs and SNPs in our trans-eQTL results than in the whole data set) (odd ratio: 45.8, Fisher's exact test pvalue = 5e-5). Two variants, rs1156058 and rs705170, were associated with a total of 14 and 7 genes in trans respectively (**Figure S4**). We also found that rs4781011, located on chromosome 16 within 5 kb of the TSS of the gene CIITA (class II, major histocompatibility complex transactivator), a gene known to activate in trans the HLA locus on chromosome 6, was a trans-eQTLs of CD74 on chromosome 5, a protein that regulates antigen presentation. This analysis shows that it is much more difficult to detect trans-eQTLs than cis-eQTLs at the same false discovery rate. Although our replication cohorts had a sample size representing only roughly 40% of the discovery cohort, we replicated approximately 50% of the trans-eQTLs attempted. This encouraging result suggests that more trans-eQTLs could be replicated with a bigger replication cohort and that our trans-eQTLs detection methodology is efficient.

**Table 1:** Top 15 trans-eQTLs (5% FDR).

SNP	gene	Probe	Chr SNP	Chr gene	rho	−log_10_(pvalue)
rs10876864	BEND4	ILMN_1740094	12	4	−0.7	128.4016
rs7192	ERG	ILMN_1768301	6	21	−0.45	43.7952
CNVR8164.1	IL37	ILMN_1697710	22	2	0.443	42.3473
rs3823342	NDUFS1	ILMN_1728810	6	2	0.435	40.6971
rs2734975	TPD52L2	ILMN_2323633	6	20	0.371	28.9582
rs13292096	LOC645688	ILMN_1772888	9	17	−0.341	24.3602
rs10902222	SHFM1	ILMN_1794505	11	7	0.341	24.3479
rs10876864	DCAF16	ILMN_1753440	12	4	−0.339	24.0678
rs6776967	STX19	ILMN_1775587	3	3	−0.332	23.0584
rs11881477	CCM2	ILMN_1784352	19	7	0.292	17.7835
CNVR2845.48	CSNK2A1	ILMN_2386355	6	20	−0.296	17.6496
rs1682809	CRISP1	ILMN_1758212	19	6	−0.286	17.1069
rs1156058	NAPSB	ILMN_2109416	1	19	−0.279	16.3070
rs1156058	CCR6	ILMN_1690907	1	6	−0.278	16.1857
CNVR6703.1	DUSP22	ILMN_1784352	16	6	0.277	16.0514

Genome-wide association studies (GWAS) found many SNPs associated with diverse phenotypes but the mechanistic link between the GWAS-SNP and the phenotype remains unclear for the vast majority of the associated SNPs. One possibility is that a GWAS-SNP affects gene expression, which then leads to the phenotype. It was previously shown that trait associated SNPs were more likely to be cis-eQTLs [18]. However, since the publication of this result, many more GWAS were performed, increasing dramatically the number of variants associated with complex traits and a much larger number of eQTLs were discovered in this study. In order to confirm that GWAS identified variants are more likely to be cis-eQTLs and to investigate if a similar relationship exists for trans-eQTLs, we accessed the catalog of published genome-wide association studies (http://www.genome.gov/gwastudies/) on 19 March 2012. 5381 SNPs reported in the catalog at that date were genotyped in our study. We looked for GWAS-SNPs overlapping eQTLs and found that 850/3 (15.8%/0.06% of the GWAS-SNPs) GWAS-SNP co-localized with variants significantly associated in cis/trans (**Table S5**) (**Table S6**). This is significantly more than using SNPs matched to the GWAS-SNPs for distance to closest gene and minor allele frequency (for cis-eQTLs, median = 585, pvalue<0.001) (for trans-eQTLs, median = 0, pvalue<0.01) (**Figure S5**). This confirms that many GWAS-SNPs are probably playing a role on disease susceptibility by affecting gene expression in cis and that trait associated SNPs are also more likely to be trans-eQTLs [19].

We next sought to determine whether trans-eQTLs were also cis-eQTLs, as this may indicate that the genes regulated in cis play a role in the regulation of the trans genes. We examined the overlap between trans-eQTLs and cis-eQTLs and found that 5 (18.5%) of the unique trans-eQTLs were also associated with gene expression in cis. This overlap is significantly greater than the overlap obtained using variants matched to the trans-eQTLs for distance to closest gene and minor allele frequency (1000 permutations, median = 0, pvalue<0.001) (**Figure S6**). We find that the cis-eQTLs of two transcription factors, BATF3 and HMX2, are associated to the most genes in trans. The cis-eQTL of BATF3, a gene involved in the differentiation of CD8α^+^ dendritic cells and IL17-producing T helper cells [20], [21], is a trans-eQTL of 14 genes, distributed on 8 chromosomes. The cis-eQTL of HMX2 is a trans-eQTL of 7 genes distributed on 4 chromosomes. HMX2 is a transcription factor directing development of inner ear and hypothalamus in mice [22] and deletion of the chromosomal region containing HMX2 in human is associated to inner ear malformations, vestibular dysfunction and hearing loss [23]. Other genes with a cis-eQTL that is also a trans-eQTL are: GNA15, a G protein, S1PR4, a G protein coupled receptor, PIDD, an effector of p53 apoptosis in mice and CRIPAK, an inhibitor of the PAK1 transcription factor. These results show that we can detect potential new functional targets of important genes in LCLs by combining cis-eQTLs and trans-eQTLs.

**Figure 3:**
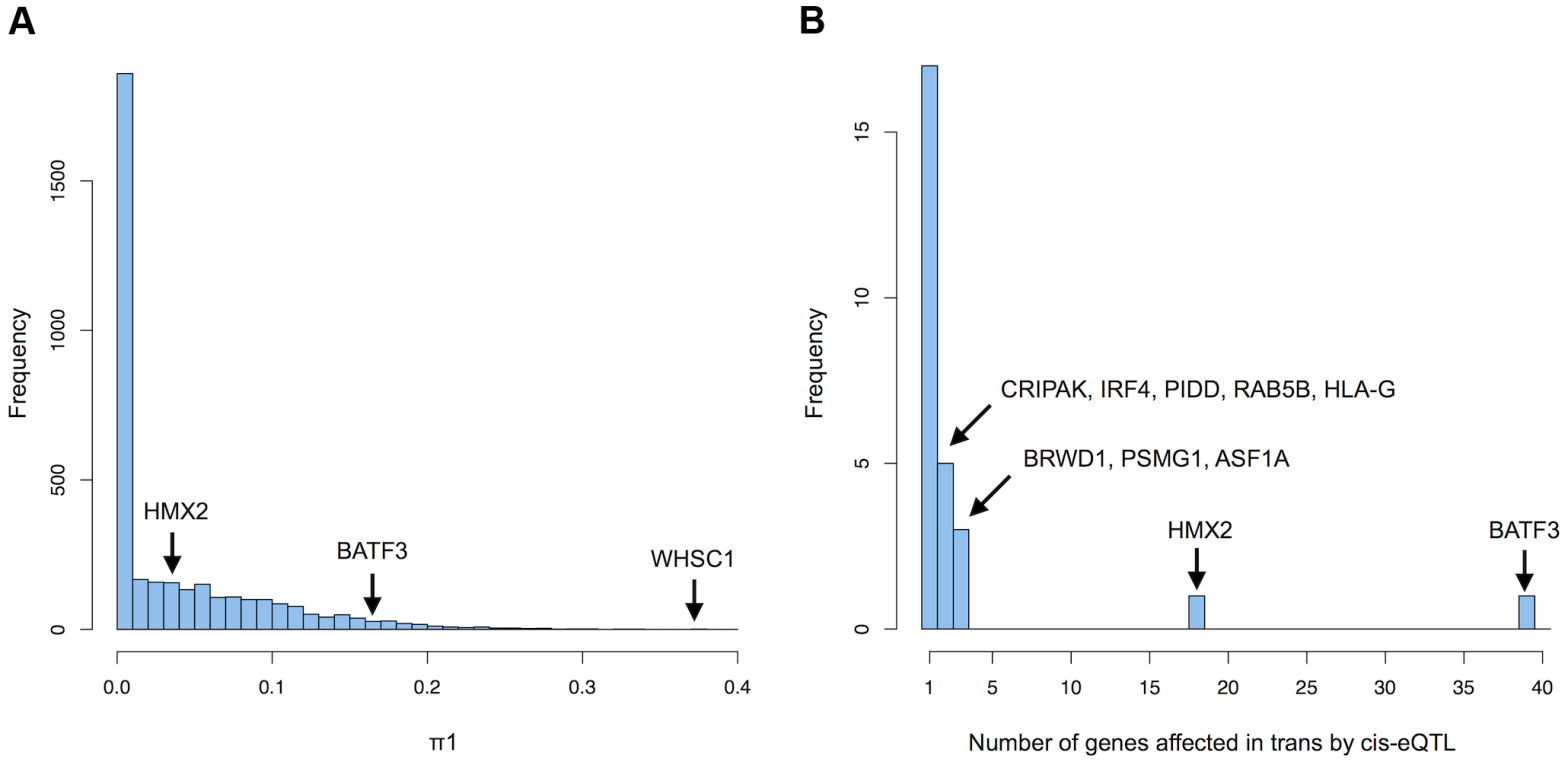
Trans-effects of cis-eQTLs. (A) Histogram of the proportion of probes (π1) affected in trans by each cis-eQTL that could be detected with a very large sample size. The cis-eQTLs of several genes is highlighted in black. (B) Histogram of the number of genes significantly affected in trans by cis-eQTLs (5% FDR) detected with 869 individuals.

In order to detect more possible functional relationships between genes regulated in cis and in trans by the same variants, we looked for the trans effects of the subset of variants that were found to be cis-eQTLs. Since we discovered 3475 variants associated to 3534 genes in cis, a trans-analysis of this subset of variants has the advantage of reducing the number of tests performed and therefore allows us to discover more trans effects of cis-eQTLs. Before investigating which cis-eQTLs are affecting which genes in trans, we first aimed to assess how many trans effects of cis-eQTLs could be detected if we had a much larger sample size. We used spearman rank correlation to test for associations between 23'935 probes, measuring the expression of 16'505 genes and all unique cis-eQTLs further than 5 MB from the TSS. We obtained approximately 23'935 trans association pvalues per cis-eQTL and computed the π1 statistics (estimate of the proportion of true positives in a pvalue distribution) on each set of pvalues, resulting in 3475 π1 estimates [16]. These estimates represent the proportion of probes that are affected in trans by the 3475 variants that are cis-eQTLs, without being able to pinpoint all individually significant effects. We observed that a large number of cis-eQTLs are affecting a large number of probes in trans (52% of the cis-eQTLs have a π1 >0) ranging from a few probes affected to up to 37.2% of the probes (median = 0.004603 corresponding to 110 probes) (**Figure 3A**). Interestingly, the variant with the most trans effects (37.2% of the probes), rs482519, is the cis-eQTL of WHSC1 (Wolf-Hirschhorn Syndrome candidate1), a histone methyltransferase. A potential explanation for this result it that variation of the level of expression of this histone methyltransferase could affect the expression of many genes by modifying chromatin accessibility. The second variant with the most trans effects (33.5% of the probes), rs2978387, is the cis-eQTL of ZNF16 (Zinc Finger Protein 16), a protein that may act as a transcription factor. The third variant with the most trans effects (32.1% of the probes), rs12196956, is the cis-eQTL of TBC1D22B (TBC1 domain family member 22B), a protein that may act as a GTPase-activating protein for Rab family protein. Furthermore, we observed a negative correlation between the strength of the cis-eQTLs and the number of probes affected in trans (spearman rho = −0.1, pvalue = 4.2e-10), suggesting that strong cis-eQTLs may be selected against in the population for genes modulating the expression of many genes. These results show that cis-eQTLs can have trans effects on many genes, which have direct consequences on regulatory network perturbations.

Although we estimated that a large number of cis-eQTLs are affecting many genes in trans, we would need a very large sample size to detect all of them at a reasonable false discovery rate. In order to assess which cis-eQTL is affecting which genes in trans, a genome-wide significance threshold was determined by permuting all expression phenotypes 1000 times (**Methods**). 92 genes had significant trans-effects due to cis-eQTLs (FDR = 5%) (**Table S4**). We replicated 31 associations (of 79 tested) in the first set of twins of the MUTHER cohort, 22 in the second set (union = 34) and 27 in GEUVADIS (of 75 tested). We discovered substantially more trans-effects of the cis-eQTLs of BATF3 and HMX2 with 39 and 18 genes regulated in trans respectively (**Figure 3B**). Other examples of cis-eQTLs with several significant trans associations include the cis-eQTL of PSMG1 (proteasome assembly chaperone 1) affecting 3 genes in trans and the cis-eQTL of BRWD1 (bromodomain and WD repeat-containing protein 1), which may be a transcriptional activator [24], also affecting 3 genes in trans. We did not find significant effects of the cis-eQTL of WHSC1, indicating that the large number of effects on gene expression have too small effect sizes to be discovered individually given our sample size. In total we found that 26 variants are cis-eQTLs of 27 genes and trans-eQTLs of 92 genes. 4 genes associated in cis to a cis/trans-eQTLs also had independent cis-eQTLs. We regressed out the effect of the main eQTLs on the trans genes expression and found that in 95% of the cases the independent eQTLs had the same allelic effect as the main eQTLs, i.e. the high expressing allele of the main eQTL has the same effect - high or low – in the trans gene as the high expressing allele of the second independent eQTL in 95% of the cases. This concordance further highlights the biological relevance of these trans eQTLs since their downstream biological effects, mediated by the modulation of the cis genes, are replicated by independent variants. 1 independents cis-eQTL (associated to HMX2) was also significantly associated to 1 gene in trans (5% FDR) and had the same allelic effect as the main eQTL. The strong concordance in allelic effects between main cis-eQTLs and independent cis-eQTLs indicate that for those 4 genes, most of the trans effects are due to variations in the level of expression of the cis gene.

We then explored whether the trans associations of the cis-eQTLs were causally due to the variation in the expression level of the cis genes. We assessed the likelihood of three different models using two methods: Bayesian networks and a causal inference test (CIT) (**Methods**) [25]. The first model (SCT) states that the variant is affecting the expression level of the cis gene, which then leads to variation in the level of expression of the trans gene. The second model (INDEP) states that the trans effect and the cis effect are independent and the third (STC) unlikely model states that the variant is affecting the level of expression of the trans gene, which then affects the level of expression of the cis gene. We observed that 100%/100%/94% of the SCT/STC/INDEP models detected by the CIT method are also detected as the best model by Bayesian networks. Conversely, 86%/33%/100% of the SCT/STC/INDEP models called by Bayesian networks were also detected as the best model by CIT. By taking the overlap of the two methods, we obtain 19 SCT, 2 STC and 49 INDEP relationships. We found causal effects (SCT) of CRIPAK on AVP, CCL5 on NPSR1, BATF3 on three genes and HMX2 on 14 genes (**Table 2**). The large representation of INDEP relationships is due to several factors. First, false positives will be called INDEP because their association is not due to the cis gene expression. As we found 92 trans associations of cis-eQTLs at a 5% FDR, we expect ∼5 INDEP relationships due to false positives. In addition, we expect 1 INDEP relationship because one cis-eQTLs is associated to two genes in cis and 2 genes in trans in total. It is unlikely that both of the cis associated genes would have causal effect on one trans associated gene leading to INDEP calls for 1 relationship. Finally, we observed that 34 INDEP associations are due to the cis-eQTL of BATF3. The INDEP relationships show that the trans gene associations are not due to the cis gene expression. However, they could be due to change in the structure of the protein if other functional variants, such as non-synonymous SNPs or splice variants, are in linkage disequilibrium with the cis-eQTLs. Alternatively, the cis-eQTLs could be affecting the expression of non-coding RNA in the vicinity of the cis genes that could drive the trans associations.

**Table 2:** Causal directed relationships between variation in level of expression of the cis gene and variation in the level of expression of the trans gene.

SNP	Cis gene	Trans gene	Cis probe	Trans probe	Variance of trans association explained by cis probe	Correlation between cis and trans probe	Correlation between cis and trans probe homozygous ref	Correlation between cis and trans probe heterozygous	Correlation between cis and trans probe homozygous alt
rs1156058	BATF3	SLC44A1	ILMN_1763207	ILMN_1700695	0.99	0.45	0.44	0.42	0.59
rs1156058	BATF3	STK17B	ILMN_1763207	ILMN_1798543	0.81	−0.23	−0.26	−0.19	−0.26
rs1156058	BATF3	SUSD1	ILMN_1763207	ILMN_1709750	0.82	−0.30	−0.28	−0.26	−0.56
rs4130791	CRIPAK	AVP	ILMN_2140700	ILMN_1811443	0.94	0.60	0.63	0.59	0.46
rs4239252	CCL5	NPSR1	ILMN_2098126	ILMN_1704206	0.98	0.45	0.42	0.45	0.58
rs705170	HMX2	CD38	ILMN_1671619	ILMN_2233783	1.00	−0.53	−0.44	−0.57	−0.60
rs705170	HMX2	NME8	ILMN_1671619	ILMN_1770940	0.87	0.33	0.26	0.33	0.26
rs705170	HMX2	DAAM1	ILMN_1671619	ILMN_1691334	0.85	0.46	0.36	0.47	0.36
rs705170	HMX2	SLC15A3	ILMN_1671619	ILMN_1787251	0.92	−0.40	−0.34	−0.39	−0.29
rs705170	HMX2	SLC47A1	ILMN_1671619	ILMN_2085862	0.86	0.34	0.29	0.31	0.34
rs705170	HMX2	DMKN	ILMN_1671619	ILMN_1798712	0.88	0.40	0.28	0.43	0.56
rs705170	HMX2	RNASE6	ILMN_1671619	ILMN_1748473	0.75	0.32	0.19	0.37	0.23
rs705170	HMX2	CDH1	ILMN_1671619	ILMN_1670325	1.00	0.64	0.58	0.63	0.68
rs705170	HMX2	USP4	ILMN_1671619	ILMN_1778319	1.00	0.46	0.42	0.44	0.40
rs705170	HMX2	GIMAP4	ILMN_1671619	ILMN_1714364	0.74	0.31	0.17	0.38	0.25
rs705170	HMX2	PTK2	ILMN_1671619	ILMN_1780533	0.98	0.58	0.50	0.60	0.48
rs705170	HMX2	RAB37	ILMN_1671619	ILMN_2255579	0.71	0.22	0.07	0.27	0.29
rs705170	HMX2	FES	ILMN_1671619	ILMN_1693650	0.81	0.30	0.21	0.34	0.16
rs705170	HMX2	GIMAP5	ILMN_1671619	ILMN_1769383	0.83	0.39	0.28	0.44	0.21

As we hypothesised that the trans-effects of some cis-eQTLs could be due to changes in the protein structure, we investigated the trans effects of 11564 non-synonymous SNPs discovered by the 1000 genome project and genotyped in the ALSPAC cohort. We used spearman rank correlation to test for associations between the 23'935 probes, measuring the expression of 16'505 genes and all non-synonymous SNPs further than 5 MB from the TSS. We first looked at large effects that could be detected given our sample size and found that 9 genes were affected in trans by non-synonymous SNPs (5% FDR) (**Table S7**). We replicated 4 associations (of 6 tested) in the first set of twin of the MUTHER cohort, 4 in the second set (union = 4) and 1 (of 7 tested) in GEUVADIS. We then looked at the global effects of non-synonymous SNPs on gene expression in trans by looking at the proportion of true positive in the distribution of the trans association pvalues for each variant using the π1 statistic [16]. We found that non-synonymous SNPs have significantly less trans effects on gene expression than cis-eQTLs (Mann-Whitney U test, pvalue = 1.8e-9) (**Figure S7**), as observed previously [4]. This result is compatible with the observation that common regulatory SNPs have more effects on complex traits and common diseases than common non-synonymous SNPs.

Finally, we explored the trans effects of the 5381 SNPs associated with complex traits and diseases in order to detect potential effects of these variants on gene expression and discovered that 66 of them are significantly associated to 10 genes in trans (5% FDR) (**Table S8**). We replicated 6 associations (of 7 tested) in the first set of twin of the MUTHER cohort, 6 in the second set (union = 6) and none in GEUVADIS (of 3 tested). For example, we found that rs11171739, which is associated to type 1 diabetes, is a trans-eQTL of DCAF16, as previously shown in monocytes [26] and is also a trans-eQTL of BEND4. We also found that rs4781011, which is associated to ulcerative colitis, is a trans-eQTL of CD74, a protein involved in immune response. rs2227139, which is associated to hematological parameters was associated in trans to ERG, which regulates hematopoiesis and the function of adult hematopoietic stem cells [27]. These results show that we can detect downstream effects of disease-associated variants, an important step to understand the relevant biological pathways in common diseases.

## Discussion

The large sample size of the ALSPAC cohort allowed us to discover that 3534 genes are affected by genetic variants in cis and 48 in trans. We found that CNVs are enriched in the best associations per gene in cis and to an even greater extent in trans. This enrichment is not surprising as CNVs are more likely to disrupt regulatory elements than SNPs due to their size [8]. This result indicates that CNVs are more likely to be causal than SNPs in genetic diseases resulting from the misregulation of gene expression. Several examples of genetic disorders, such as aniridia, sex-reversal and holo-prosencephaly are already known to be caused by duplications or deletions of CNVs located in non-coding regions of developmental genes [28]. We found that SNPs associated with complex traits and common diseases are more likely to be cis and trans-eQTLs than matched variants. Although some of these overlaps might be coincidental [3], these results further confirm that a significant fraction of trait associated SNPs are acting at the gene expression level.

We observed that many eQTLs detected in skin and adipose tissues could not be detected in LCLs irrespectively of the sample size, showing that a significant fraction of eQTLs is tissue specific. Therefore, eQTL studies in many different tissues are needed in order to map all regulatory variants in the human genome and understand their precise tissue specific effect, a necessary step to understand why a specific tissue becomes the “disease” tissue and not other tissues.

We estimated that 52% of the cis-eQTLs have trans-effects on gene expression ranging from a few probes to up to 37.2% of the probes. The large number of trans-effects of cis-eQTLs is concordant with the fact that on average 65% of the heritability of gene expression is trans to the gene in LCLs [2]. As we can detect only a minority of these effects at a reasonable false discovery rate with our relatively large sample size, it indicates that most of the trans-effects of the cis-eQTLs are of small effect sizes. If complex traits and common diseases have the same underlying architecture as gene expression, a substantial part of the missing heritability will then be due to many common variants of very small effect sizes.

Using Bayesian networks and causal inference tests [25], we could detect 19 cases where a variant affects the expression of a gene in cis that is causally affecting the expression of a gene in trans (14 due to the cis-eQTL of HMX2). For example, the level of expression of CRIPAK, a protein implicated in cytoskeleton remodelling and influencing PAK1 mediated estrogen transactivation activity [29], is causally affecting the level of expression of AVP (arginine vasopressin), a hormone with anti-diuretic effects on the kidney and affecting social behaviour [30]. Taken together, these results show that population based strategies allow to detect important relationships between genes. Ultimately, this type of approach performed with larger sample sizes will allow us to uncover the cascade of events that lead a disease associated variants to the disease phenotype.

## Methods

### Ethical approval

Ethical approval for the study was obtained from the ALSPAC Ethics and Law Committee and the Local Research Ethics Committees.

### Study sample

ALSPAC is a prospective birth cohort which recruited pregnant women with expected delivery dates between April 1991 and December 1992 from Bristol UK. 14,541 pregnant women were initially enrolled with 14,062 children born. Detailed information on health and development of children and their parents were collected from regular clinic visits and completion of questionnaires. A detailed description of the cohort is available on our website (http://www.bristol.ac.uk/alspac/researchers/) and has been published previously [31]. Please note that the study website contains details of all the data that is available through a fully searchable data dictionary (http://www.bris.ac.uk/alspac/researchers/data-access/data-dictionary/).

DNA has been extracted as described previously from blood samples collected from cord blood at research clinics [32]. Lymphoblastoid cell lines were established by transforming lymphocytes from blood samples taken when the study participants were 9 years old, with Epstein Barr Virus.

### Genotyping data

ALSPAC individuals were genotyped using the Illumina HumanHap550 quad genome-wide SNP genotyping platform by 23andMe subcontracting the Wellcome Trust Sanger Institute, Cambridge, UK and the Laboratory Corporation of America, Burlington, NC, USA. Markers with <1% MAF, >5% missing genotypes or which failed an exact test of Hardy-Weinberg equilibrium (P<5×10−7) were excluded from further analysis. Any individuals who did not cluster with the CEU individuals in multidimensional scaling analysis, who had >3% missing data, minimal or excessive heterozygosity (>33% or <31%), evidence of cryptic relatedness (>10% IBD) or incorrect gender assignments were excluded from further analysis. After data cleaning 315,807 SNPs were left. Imputation was carried out using MACH 1.0.16, Markov Chain Haplotyping [33], [34], using CEPH individuals from phase 2 of the HapMap project as a reference set. Imputed markers with imputation quality r^2^<0.8, with MAF<1% or which failed an exact test of Hardy-Weinberg equilibrium (P<5×10−7) were excluded resulting in a total of 2'290'057 high quality SNPs. The CNVs were genotyped using a targeted Agilent 105K CGH array. The design of the array and the methodology for analyzing the array data was previously described in details [35].

### Gene expression data

LCL's from unrelated individuals were grown under identical conditions and cells frozen in RNAlater. RNA was extracted using an RNeasy extraction kit (Qiagen) and was amplified using the Illumina TotalPrep-96 RNA Amplification kit (Ambion). Expression profiling of the samples, each with two technical replicates, were performed using the Illumina Human HT-12 V3 BeadChips (Illumina Inc) including 48,804 probes where 200 ng of total RNA was processed according to the protocol supplied by Illumina. Raw data was imported to the Illumina Beadstudio software and probes with less than three beads present were excluded. Log2 - transformed expression signals were then normalized with quantile normalization of the replicates of each individual followed by quantile normalization across all individuals. We restricted our analysis to 23'935 probes tagging genes annotated in Ensembl. Principal component analysis was performed on 931 individuals. 62 individuals with principal component 1 or 2 greater than one standard deviation of the population were excluded from further analysis. Raw expression data are available upon request at http://www.bristol.ac.uk/alspac/researchers/data-access/policy/.

### eQTL analysis

All eQTL analysis were performed at the single variant level and assumed an additive model. We used spearman rank correlation to test for association between probe expression and genotype. For the cis-analysis, we limited the variants tested to variants present in a 2 MB window surrounding the transcription start site of the gene and we filtered out probes containing SNPs with minor allele frequency >1% according to the 1000 genomes project dataset [36]. To assess significance, we permuted all expression probes 1000 times and kept the best pvalue per gene after each permutation. For each gene, we ranked the permutation pvalues and assessed whether a variant in the non-permuted data had a lower association pvalues than the permutation threshold considered. We then computed the false discovery rate associated with the permutation threshold and subsequently selected the permutation threshold that provides a 5% false discovery rate.

For the trans analysis, we tested all variants except variants present in a 5 MB window surrounding the transcription start site. In order to remove false positives, we excluded probes mapping to multiple locations according to ReMOAT [37]. To assess significance, we permuted 1000 times 288 random probes, each corresponding to one gene. As each probe is tested by approximately the same number of SNPs and as we used spearman rank correlation, which is robust to outliers, we treated our permutations as if we had permuted one probe 288'000 times. We combined all pvalues obtained from the permutations (288*1000), ranked them and increased the genome-wide pvalue threshold until we reached a 5% false discovery rate (corresponding to a pvalue of 9.5e-11).

For the trans analysis of cis-eQTLs, we tested all unique cis-eQTLs except variants present in a 5 MB window surrounding the TSS. In order to remove false positives, we excluded probes mapping to multiple locations according to ReMOAT [37]. To assess significance, we permuted all expression probes 1000 times. As for the trans analysis of all variants, we combined all pvalues obtained, ranked them and increased the genome-wide pvalue threshold until we reached a 5% false discovery rate (corresponding to a pvalue of 7.6e-8).

For the trans analysis of non-synonymous SNPs and SNPs associated to complex traits and diseases, we tested all SNPs except variants present in a 5 MB window surrounding the TSS. In order to remove false positives, we excluded probes mapping to multiple locations according to ReMOAT [37]. To assess significance, we permuted 1000 random probes, corresponding to 1000 genes, 10000 times. As for the other trans analysis, we combined all pvalues obtained, ranked them and increased the genome-wide pvalue threshold until we reached a 5% false discovery rate (corresponding to a pvalue of 2.0e-9 for non-synonymous SNPs and 5.4e-10 for SNPs associated to complex traits and diseases).

### Conditional regression

For each gene with an eQTL, we performed linear regression of the best variant on the standard normalized probe expression and kept the residuals. We repeated the association analysis on the residuals using spearman rank correlation and kept any SNPs passing the gene-based permutation threshold obtained during the initial association analysis. We repeated this procedure regressing out all previous best associations until no variants were significant.

### Heritability explained by cis-eQTLs

For each gene with cis-eQTL(s), we computed the variance explained (r^2^) by the best cis-eQTLs or all independent cis-eQTLs on the standard normalized probe expression using the lm() function in R. We then obtained the heritability explained by dividing the heritability of the probe with the variance explained by the cis-eQTL(s). If the variance explained by the cis-eQTL(s) was greater than the heritability estimate of the probe, the heritability explained was set to 1.

### Matched SNPs in enrichment analysis

We matched each significant variant (cis-eQTLs, trans-eQTLs or GWAS SNPs) with a variant with the same minor allele frequency in our data set (±1%) and distance to the closest gene (±2 kb).

### Causal models

Bayesian networks (BN) are directed acyclic graphs where nodes represent random variables and edges represent the conditional dependences among nodes. The direction of the edges between two nodes can be interpreted as causal relationships and allowed to infer causality in genetics studies previously [38]–[40].

Likelihood methods are commonly used to compare different BN and estimate the most likely—that is, the set of causal relationships among the different variables that better agrees with the data. In a BN, every node is associated with a probability distribution and, together with the conditional dependencies represented by the edges, forms the join probability distribution of the network. BN satisfy the local Markov property—that is, each variable is conditionally independent of its non-descendants given its parent variables. The Markov property allows the decomposition of the joint probability distribution of the network into a set of local distributions, which allows to easily calculate the likelihood of a given BN.

We used the R package bnlearn [41] to calculate the maximum likelihood of three different networks that we defined using eQTLs as anchors. In the first network (SCT), we fixed the first node as the eQTL genotype with a forward directional edge to the second node (standard normalized cis gene expression) and a second forward directional edge starting from the second node to the third node (standard normalized trans gene expression). For the second network (STC), we fixed the first node as the eQTL genotype with a forward directional edge to the second node (standard normalized trans gene expression) and a second forward directional edge starting from the second node to the third node (standard normalized cis gene expression). For the third network (INDEP), we fixed the first node as the eQTL genotype with a forward directional edge to a node representing the standard normalized cis gene expression and a second forward directional edge starting from the first node to a node representing the standard normalized trans gene expression.

Different networks often have different complexities and it is common to use a score that takes into account the network complexity instead of the raw likelihood to compare different networks. We used the Akaike Information Criterion (AIC) score (AIC = 2k-2ln(L), where k is the number of parameters (5 for all models in our case) and L is the maximum likelihood) to compare our networks. To compare how good is a network compared to another, we used the relative likelihood of one network against the other. If we have two networks, N1 and N2 and AIC(N1)≤AIC(N2), then the relative likelihood of N2 with respect to N1 is defined as: exp((AIC(N1)–AIC(N2))/2). We kept only networks where the best model was at least ten times more likely than the second best model. In order to have high confidences in our calls, we required that the Causal Inference Test (CIT), described previously [25], also calls the same model as the most likely. The CIT is a semi-parametric method that tests a series of conditions and then provides p-values for the SCT and STC models. If none of them has a pvalue<0.05, it makes a call for the INDEP model, and if both of them are significant it makes no call. In order to take into account multiple testing with the CIT method and to reduce the number of networks resulting in a “no call” by the CIT, we used Bonferroni corrected pvalues for model calling instead of the nominal pvalue of 0.05.

## Supporting Information

Figure S1 Distance of cis-eQTLs to transcriptional start site with respect to the strength of the associations (−log10 pvalue).
(TIFF)

Figure S2 Number of genes with a cis-eQTL discovered in function of sample size (black). False discovery rate associated with the number of discoveries is shown in red.
(TIFF)

Figure S3 Histogram of number of independent cis-eQTLs per gene.
(TIFF)

Figure S4 Number of genes regulated in trans per trans-eQTL.
(TIFF)

Figure S5 Histograms of the number of overlaps between eQTLs in cis (A) and in trans (B) and random SNPs matched with GWAS-SNPs for distance to closest gene and minor allele frequency repeated 1000 times with a different set of matched SNPs. The red bar represent the 99% quantile of the distributions.
(TIFF)

Figure S6 Histogram of the number of overlaps between cis-eQTLs and random SNPs matched with trans-eQTLs for distance to closest gene and minor allele frequency repeated 1000 times with a different set of matched SNPs.
(TIFF)

Figure S7 Boxplot of proportion of probes affected in trans for each non-synonymous SNPs (left) and each cis-eQTL (right) measured using the π1 statistic on the pvalue distribution of the trans associations.
(TIFF)

Table S1 Significant cis-eQTLs (5%FDR).
(XLSX)

Table S2 eQTL discovery results.
(XLSX)

Table S3 Significant trans-eQTLs (5%FDR).
(XLSX)

Table S4 Significant trans effects of cis-eQTLs (5%FDR).
(XLSX)

Table S5 Co-localization of variants associated to complex traits and diseases with variants significantly associated to gene expression in cis (5%FDR).
(XLSX)

Table S6 Co-localization of variants associated to complex traits and diseases with variants significantly associated to gene expression in trans (5%FDR).
(XLSX)

Table S7 Significant trans effects of non-synonymous SNPs (5%FDR).
(XLSX)

Table S8 Significant trans effects of variants associated to complex traits and diseases (5%FDR).
(XLSX)

Text S1 Methodology used to detect and remove probable false positive trans-eQTLs due to mismapped CNVs.
(DOCX)
