# Supplementary figures and images for: Cis and Trans Effects of Human Genomic Variants on Gene Expression
Source: PLoS Genet. 2014 Jul 10;10(7):e1004461. doi: 10.1371/journal.pgen.1004461 (PMC4091791; doi:10.1371/journal.pgen.1004461)

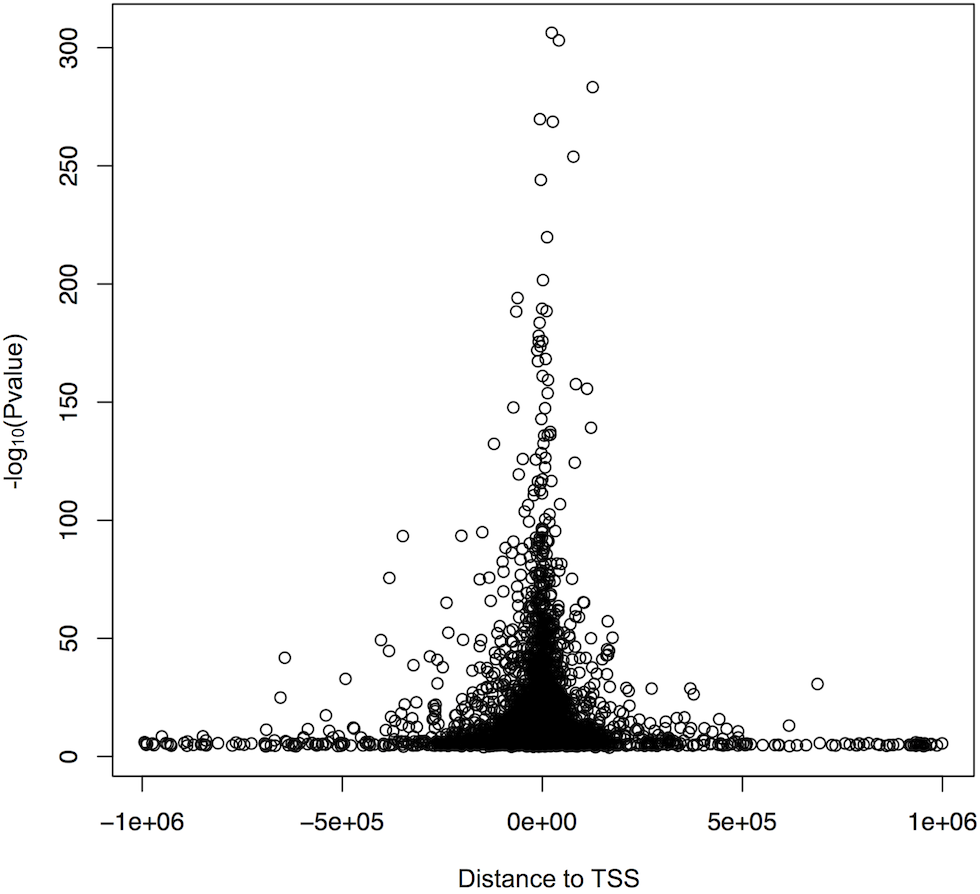

Supplement: Figure S1 — Distance of cis-eQTLs to transcriptional start site with respect to the strength of the associations (−log10 pvalue). (TIFF) [file pgen.1004461.s001.tiff]

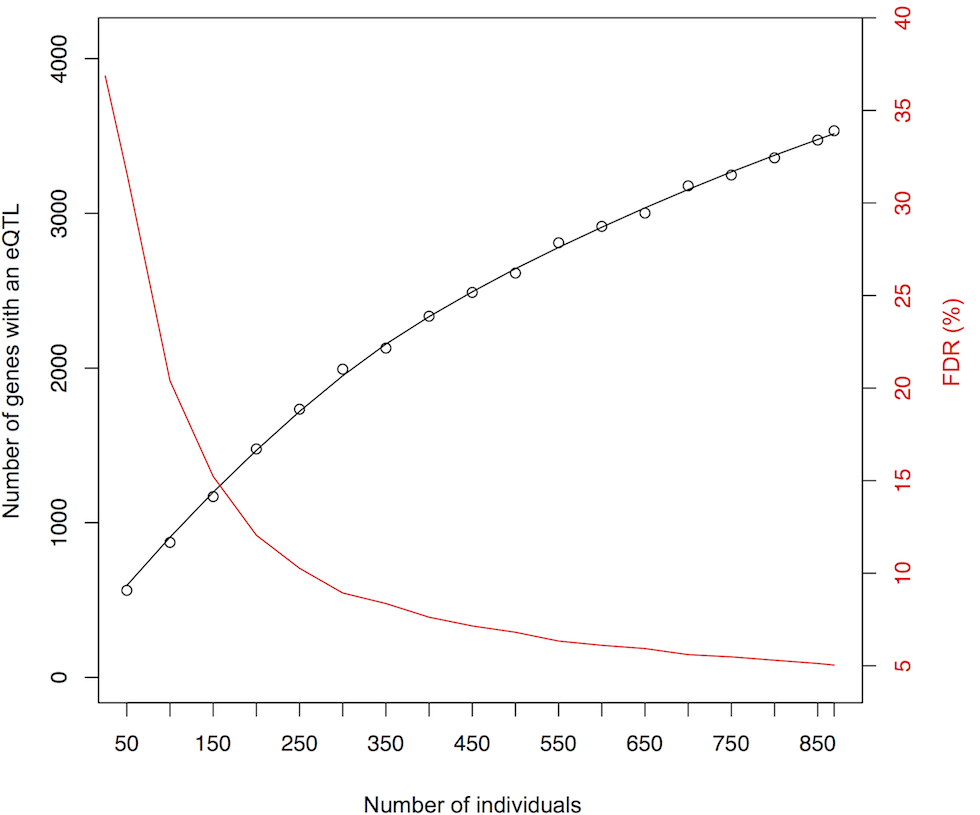

Supplement: Figure S2 — Number of genes with a cis-eQTL discovered in function of sample size (black). False discovery rate associated with the number of discoveries is shown in red. (TIFF) [file pgen.1004461.s002.tiff]

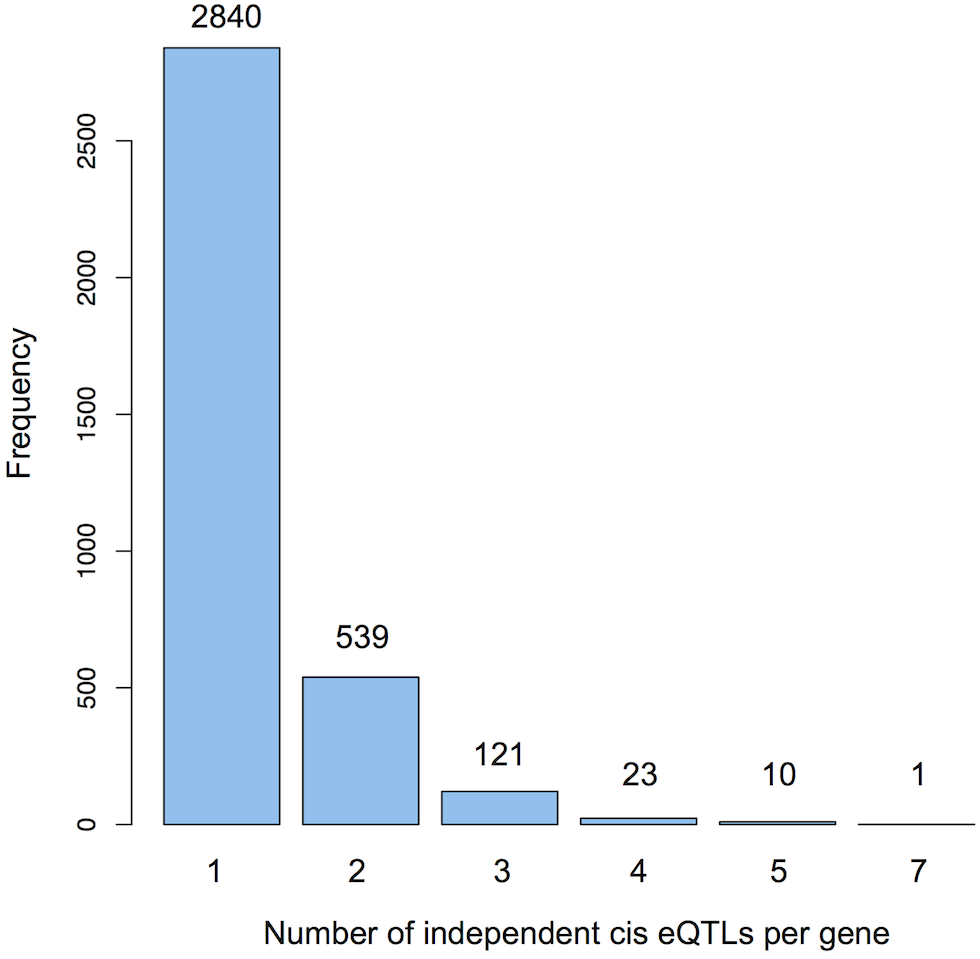

Supplement: Figure S3 — Histogram of number of independent cis-eQTLs per gene. (TIFF) [file pgen.1004461.s003.tiff]

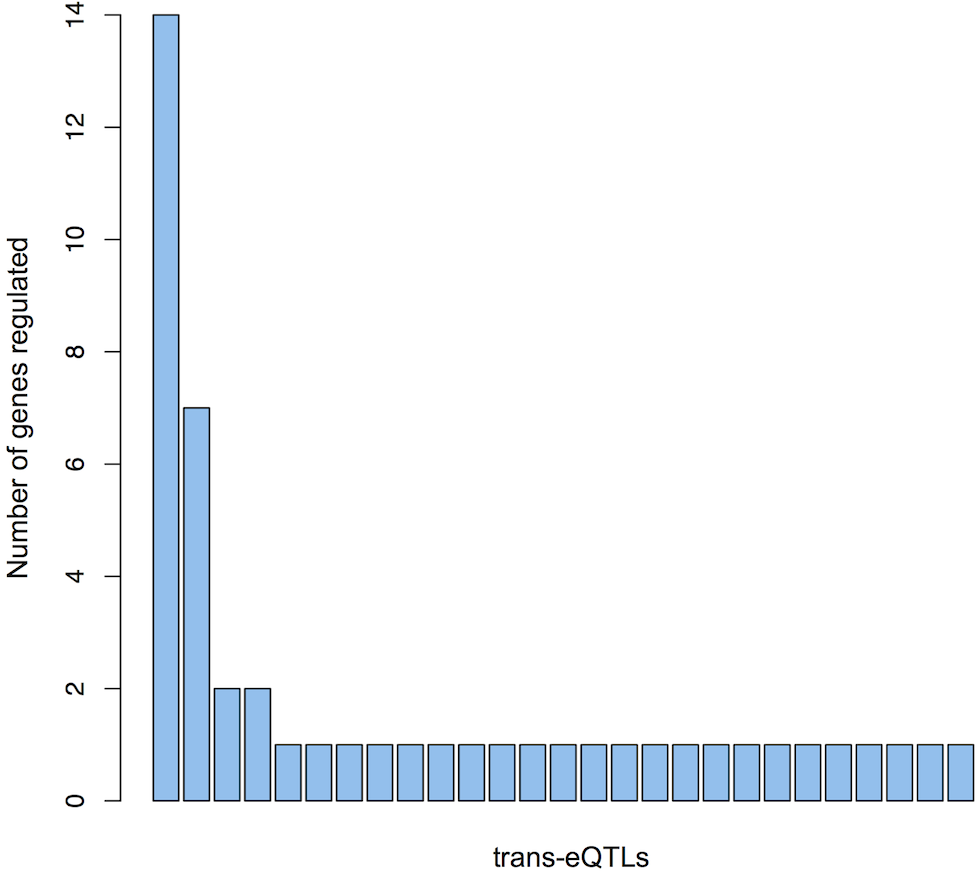

Supplement: Figure S4 — Number of genes regulated in trans per trans-eQTL. (TIFF) [file pgen.1004461.s004.tiff]

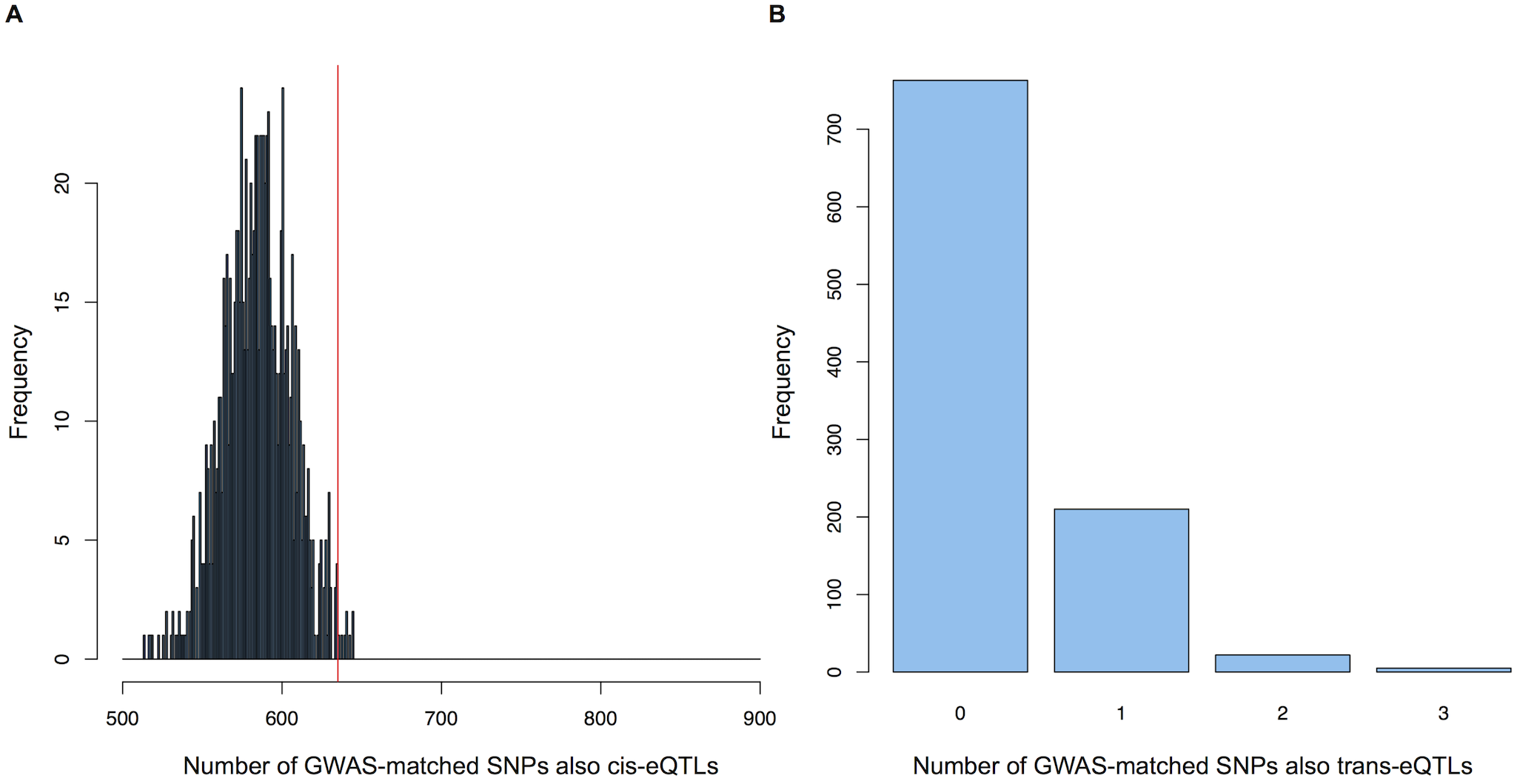

Supplement: Figure S5 — Histograms of the number of overlaps between eQTLs in cis (A) and in trans (B) and random SNPs matched with GWAS-SNPs for distance to closest gene and minor allele frequency repeated 1000 times with a different set of matched SNPs. The red bar represent the 99% quantile of the distributions. (TIFF) [file pgen.1004461.s005.tiff]

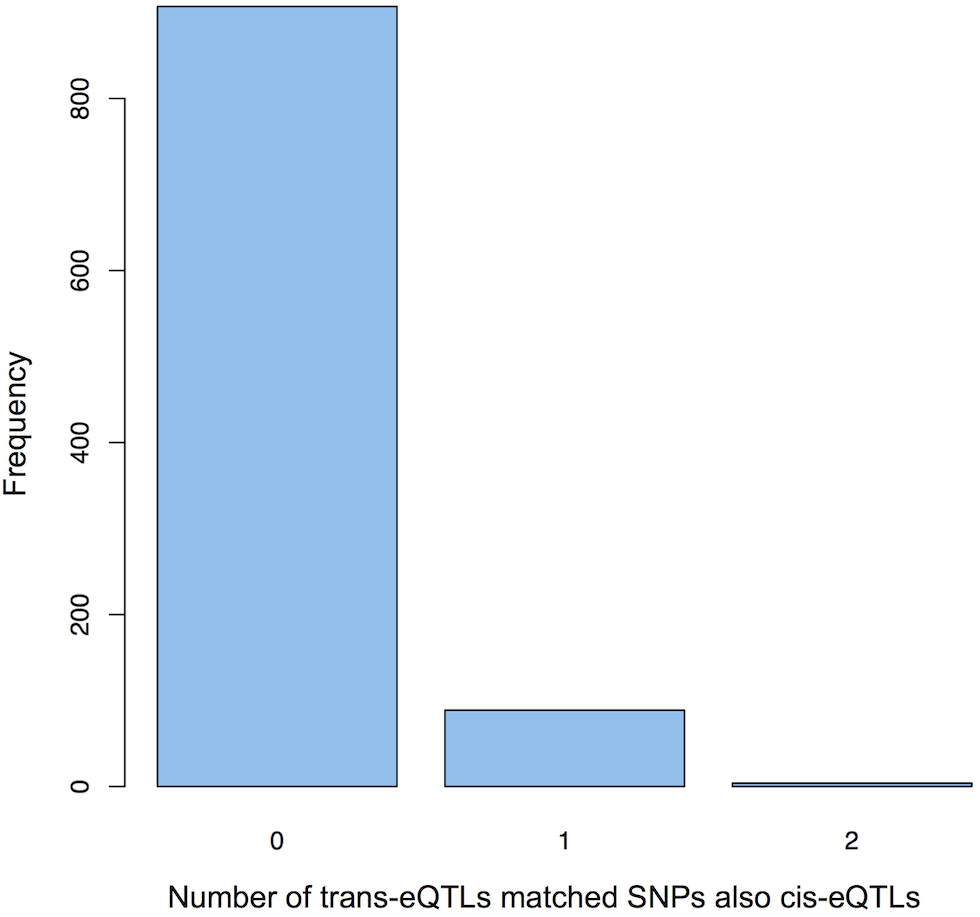

Supplement: Figure S6 — Histogram of the number of overlaps between cis-eQTLs and random SNPs matched with trans-eQTLs for distance to closest gene and minor allele frequency repeated 1000 times with a different set of matched SNPs. (TIFF) [file pgen.1004461.s006.tiff]

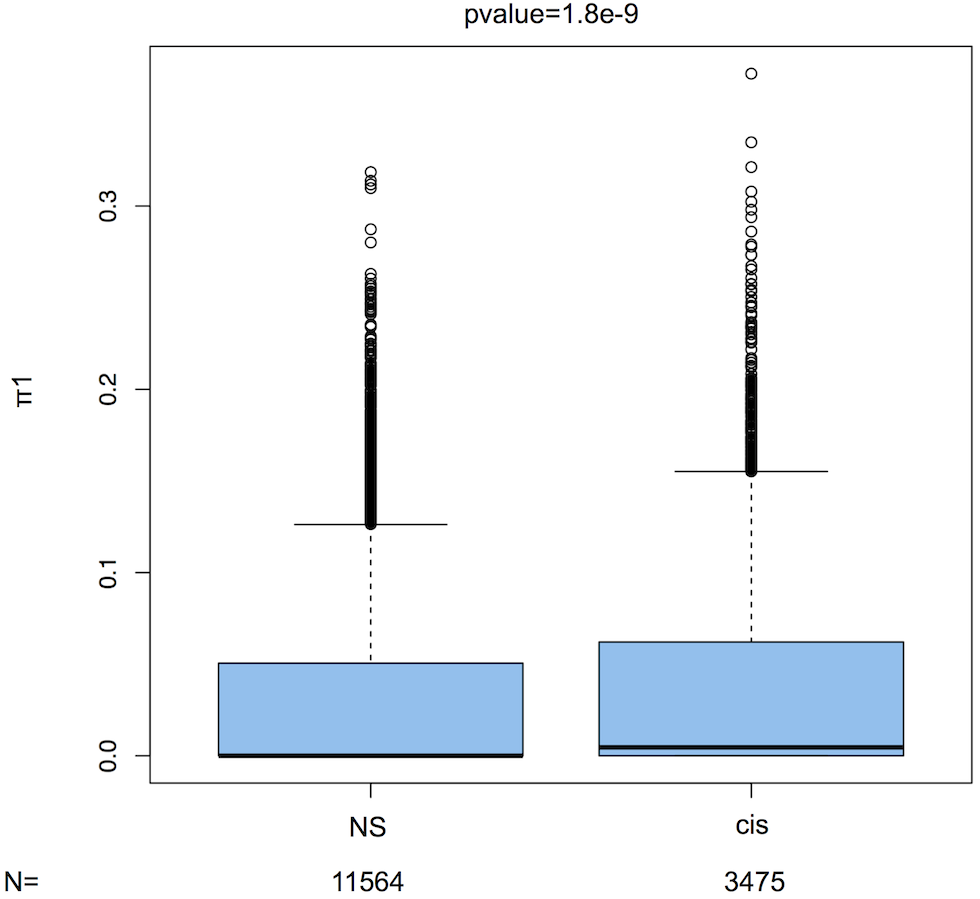

Supplement: Figure S7 — Boxplot of proportion of probes affected in trans for each non-synonymous SNPs (left) and each cis-eQTL (right) measured using the π1 statistic on the pvalue distribution of the trans associations. (TIFF) [file pgen.1004461.s007.tiff]
