# Supplementary material for: Cis and Trans Effects of Human Genomic Variants on Gene Expression
Source: PLoS Genet. 2014 Jul 10;10(7):e1004461. doi: 10.1371/journal.pgen.1004461 (PMC4091791; doi:10.1371/journal.pgen.1004461)
Supplement: Text S1 — Methodology used to detect and remove probable false positive trans-eQTLs due to mismapped CNVs. (DOCX) [file pgen.1004461.s016.docx]

***Supplementary method***

We initially discovered that some CNVs had very low association pvalues with gene expression in trans. In order to assess whether these associations were true positives, we performed correlation analysis for significant trans-SNPs and significant trans-CNVs with all other SNPs genome-wide. We found that all SNPs were correlated with SNPs close to their positions while some CNVs appeared to be correlated to SNPs located on a different chromosome than their supposed position. Some of the SNPs that were correlated to the CNVs were cis-eQTLs of the same trans gene. Therefore, in order to remove probable false trans signal due to mismapping of CNVs, we removed all trans-associations between a CNV and a gene if the gene is located on the same chromosome as the CNV of if the gene is located on a chromosome with SNPs correlated with the CNV with r^2^ >0.1.

We also discarded association of CNVs (CNVR2664.1 and CNVR3427.3) that had an imbalance in copy number between males and females as they probably represent false trans association to differentially expressed genes between males and females. In addition theses CNVs were not found to be trans-eQTLs when repeating the trans-analysis after sex-stratification.

***Examples of CNVs association that were excluded***


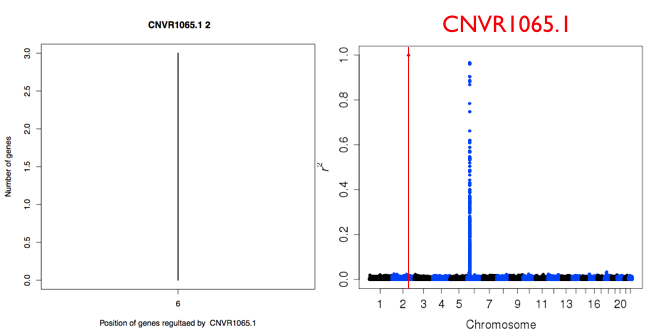


All associations of CNVR1065.1, originally located on chromosome 2, were excluded as it is correlated to SNPs on chromosome 6 that are cis-eQTLs of the trans genes. In addition no SNPs in a 2MB window surround it original position capture the trans effect. (left panel, distribution of genes chromosomes that have the CNV as “trans”-eQTL, right panel, correlation between the CNV and all SNPs genome-wide)


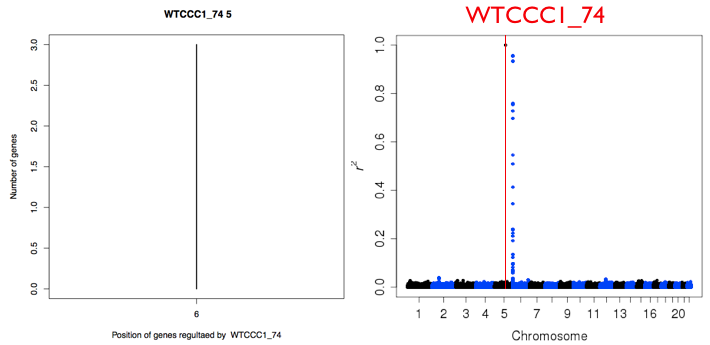
 All associations of WTCCC1_74, originally located on chromosome 5, were excluded as it is correlated to SNPs on chromosome 6 that are cis-eQTLs of the trans gene. In addition no SNPs in a 2MB window surround it original position capture the trans effect. (left panel, distribution of genes chromosomes that have the CNV as “trans”-eQTL, right panel, correlation between the CNV and all SNPs genome-wide)


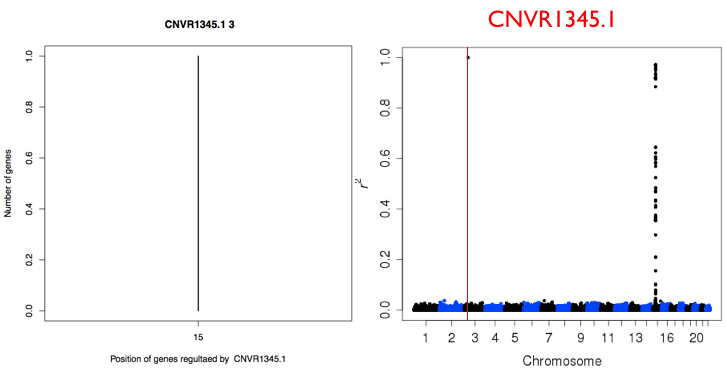


All associations of CNVR1345.1, originally located on chromosome 3, were excluded as it is correlated to SNPs on chromosome 15 that are cis-eQTLs of the trans gene. In addition no SNPs in a 2MB window surround it original position capture. (left panel, distribution of genes chromosomes that have the CNV as “trans”-eQTL, right panel, correlation between the CNV and all SNPs genome-wide)


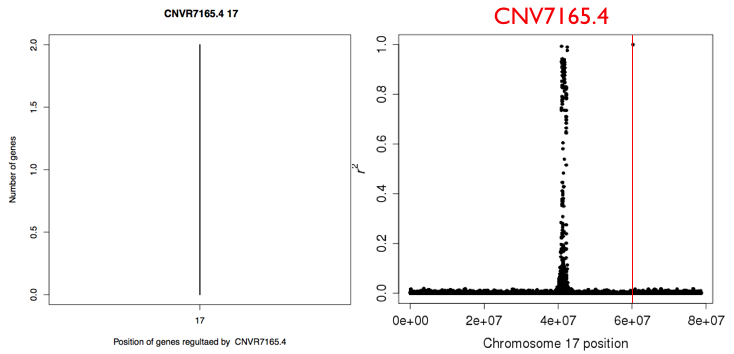


All associations of CNV7165.4 were excluded, as it seems that it is in reality located 20MB away from its original position. SNPs correlated with the CNV are cis-eQTLs of the trans gene. In addition no SNPs in a 2MB window surround its original position capture the trans effects. (left panel, distribution of genes chromosomes that have the CNV as “trans”-eQTL, right panel, correlation between the CNV and all SNPs genome-wide)


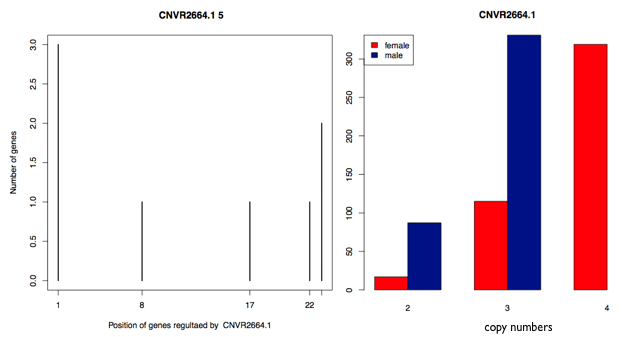
All associations of CNVR2664.1 were discarded, as CNVR2664.1 is imbalanced between males and females, leading to false trans associations to genes differentially expressed between males and females.
